# Central Venous Pressure Referencing in the Lateral Position: Comparison With Direct Right Atrial Pressure in Mechanically Ventilated ICU Patients

**DOI:** 10.1111/aas.70280

**Published:** 2026-06-12

**Authors:** Carl Sjödin, Jonatan Oras, Lotta Johansson, Per Persson, Sepideh Olausson, Jacob Holmqvist, Alexandru Ille, Nevio Vidovic, Carl Johan Svensson

**Affiliations:** ^1^ Region Västra Götaland, Sahlgrenska University Hospital Department of Anaesthesiology and Intensive Care Gothenburg Sweden; ^2^ Institute of Health and Care Sciences, Institute of Health and Care Sciences at the Sahlgrenska Academy University of Gothenburg Gothenburg Sweden; ^3^ Department of Anaesthesiology and Intensive Care Medicine, Institute of Clinical Sciences at the Sahlgrenska Academy University of Gothenburg Gothenburg Sweden; ^4^ Region Västra Götaland, Sahlgrenska University Hospital Department of Medical Physics and Biomedical Engineering Gothenburg Sweden

**Keywords:** body‐position, central venous pressure, central venous pressure measurement, critical illness, right atrial pressure, right atrial pressure measurement

## Abstract

**Background:**

Right atrial pressure (RAP) is a key determinant of venous return and reflects right‐sided filling pressures in critically ill patients. Central venous pressure (CVP) is commonly used as a surrogate, but its accuracy depends on appropriate transducer levelling. While reference points have been evaluated in supine positions, evidence is lacking for the lateral position, despite its frequent use in intensive care. Furthermore, it remains unclear whether lateral positioning alters RAP itself.

**Methods:**

In this prospective observational study, 16 sedated, mechanically ventilated ICU patients with central venous catheters were included. RAP was measured using a solid‐state catheter positioned in the mid‐right atrium, confirmed by waveform analysis and echocardiography. Simultaneous CVP was recorded with the transducer levelled 5 cm below the mid‐sternum. Measurements were obtained at end‐expiration in supine, 45° left lateral, and 45° right lateral positions. Agreement between RAP and CVP was assessed using Bland–Altman analysis, intraclass correlation coefficients, and predefined clinical thresholds. Stepwise hydrostatic adjustments were applied to identify optimal reference levels.

**Results:**

In the right lateral position, CVP referenced 5 cm below the sternum closely approximated RAP (bias −0.4 mmHg; limits of agreement −3.2 to +2.4; ICC 0.85). In the left lateral position, the same reference point systematically overestimated RAP (bias −2.2 mmHg; limits of agreement −5.0 to +0.7; ICC 0.58). Adjustment to approximately 2 cm below the sternum minimised bias (−0.1 mmHg) and improved agreement (ICC 0.82). RAP increased in the right lateral position compared with both supine (Δ +2.9 mmHg, *p* < 0.001) and left lateral (Δ +2.8 mmHg, *p* < 0.001) positions, while no difference was observed between supine and left lateral positions (*p* = 0.78).

**Conclusion:**

In mechanically ventilated ICU patients, central venous pressure accuracy was position‐dependent, and optimal external reference levels differed between lateral positions. Right atrial pressure increased in the right lateral position, suggesting that lateral positioning influences cardiopulmonary physiology beyond hydrostatic measurement effects alone.

**Trial Registration:**

https://clinicaltrials.gov/study/NCT06705374, registration date 30 April 2024

**Editorial Comment:**

The study showed that central venous pressure systematically overestimated right atrial pressure in mechanically ventilated patients placed in the left lateral position. This means that preload and venostasis can be misinterpreted in this position. The influence of increased right atrium pressure on venous return and cardiac output during left lateral positioning in mechanically ventilated patients requires further studies before the clinical relevance can be assessed.

## 1 Background

Accurate assessment of venous return is fundamental to evaluating haemodynamic status in critically ill patients. Venous return depends on the pressure gradient between mean systemic filling pressure and right atrial pressure (RAP), divided by venous resistance [1]. Thus, precise measurement of RAP is essential for detecting subtle changes that may influence venous return and, consequently, cardiac output [2].

RAP reflects right ventricular preload and is influenced by cardiac function, circulating blood volume, and both intrathoracic and intra‐abdominal pressures [3]. It also represents the back pressure to extrathoracic organs [4], making it a critical parameter for assessing organ perfusion. Elevated RAP, often indicative of venous congestion, has been associated with adverse outcomes, including acute kidney injury and increased mortality in septic, perioperative, and critically ill populations [5, 6, 7, 8].

In clinical practice, central venous pressure (CVP) is widely used as a surrogate for RAP. When measured through a central venous catheter in the superior vena cava or right atrium, CVP closely approximates RAP, provided there is no obstruction between the catheter tip and the atrium [9]. However, accuracy depends critically on proper transducer levelling relative to an external anatomical landmark [10].

Although levelling strategies have been examined in the supine position, little evidence guides reference point selection in the lateral position, despite prolonged use of lateral positioning in critically ill patients for pressure injury prevention, respiratory management, and routine nursing care [11, 12]. Moreover, little is known about how lateral positioning itself alters RAP compared with supine, though such effects likely influence pressure interpretation.

The primary aim of this study was to evaluate the agreement between central CVP, measured with the transducer levelled 5 cm below the mid‐sternum, and directly measured RAP in the left and right lateral positions. Secondary aims were (i) to determine whether an alternative levelling point could minimise systematic bias, and (ii) to describe changes in RAP between supine and lateral positions, thereby clarifying how body orientation influences intracardiac pressures in critically ill patients.

## 2 Methods

### 2.1 Study Design and Ethical Approval

This prospective observational study was conducted between April 2024 and December 2024 in a 16‐bed general intensive care unit (ICU) and an 8‐bed neuro‐ICU at Sahlgrenska University Hospital, Gothenburg, Sweden. The protocol was approved by the Swedish Ethical Review Authority (Dnr: 2023‐06085‐02) and registered at ClinicalTrials.gov (NCT06705374). Written informed consent was obtained from patients whenever possible; for those lacking capacity, presumed wish was initially provided by next of kin, with deferred patient consent when feasible.

This study represents a prespecified analysis of a registered prospective trial (ClinicalTrials.gov NCT06705374) investigating the effect of body position on right atrial pressure and central venous pressure measurements. The full protocol included haemodynamic assessments in supine, semi‐recumbent, Trendelenburg, and lateral positions. Analyses of the supine, semi‐recumbent, and Trendelenburg positions are reported separately; the present manuscript focuses specifically on the left and right lateral positions.

### 2.2 Participants

Inclusion criteria were age ≥ 18 years, controlled mechanical ventilation, and presence of an arterial line and a four‐ or five‐lumen central venous catheter (CVC) inserted via the jugular or subclavian vein.

Exclusion criteria were severe circulatory or respiratory instability, positive end‐expiratory pressure (PEEP) > 14 cmH_2_O, or intolerance to positional changes.

### 2.3 Procedures

A solid‐state catheter (Micro‐Cath, Millar Inc., TX, USA) was advanced through a medial CVC lumen into the right atrium. Placement was guided by pressure waveforms and confirmed by transthoracic echocardiography (Figures S1 and S2); chest radiography was used in the first two patients but subsequently deemed unnecessary. CVP was measured via the distal CVC lumen with a disposable pressure transducer (Xtrans, CODAN, Germany). RAP, CVP, and arterial pressures were continuously recorded (Millar TC‐510 and Philips IntelliVue MX800) at 125 Hz using Biopac AcqKnowledge 4.2. After completion of the measurements, the solid‐state catheter was removed, flushed with NaCl, and exposed to atmospheric pressure. The residual zero‐offset was recorded as a quality‐control measure (Figure S3).

Patients were sedated to Richmond Agitation–Sedation Scale (RASS) –4 to −5 and ventilated in pressure‐controlled mode with tidal volume 6–8 mL/ideal kg body weight. FiO_2_ and PEEP were set by the treating clinician and maintained during the measurement protocol. Doses of sedatives and vasopressors were kept constant. Because no validated reference level exists for the 45° lateral position, an anatomically plausible starting point was derived from CT measurements of right atrial depth relative to the mid‐sternum in five adult individuals of varying body size and sex. CT images were geometrically rotated to simulate a 45° lateral tilt under the assumption of a rigid thorax. The mean projected depth approximated 5 cm below the mid‐sternum and was used as the initial reference level.

In the right lateral position, additional intermediate levels between 3 and 5 cm below the mid‐sternum were not evaluated because the 5‐cm reference level already showed smaller bias than the 3‐cm level. In the left lateral position, an additional 2‐cm level was evaluated to identify the reference level with the smallest bias.

CVP measurements were obtained only in the 45° left lateral and 45° right lateral positions. Patients were first positioned in the 45° left lateral orientation using a positioning pillow, and then repositioned to 45° right lateral after completion of the left lateral recordings. A standardised rest period of 3 min was observed after each repositioning before pressures were recorded. RAP (and CVP where applicable) was measured simultaneously at end‐expiration from stable waveform segments using mean values derived from continuous high‐resolution recordings. Averaging stable waveform segments was chosen to minimise susceptibility to transient waveform disturbances and respiratory variability. Pulse pressure (PP) was derived from the Biopac arterial pressure waveform and averaged over three consecutive beats at end‐expiration. In one patient with atrial fibrillation, PP was not analysed because marked beat‐to‐beat variability precluded a representative end‐expiratory value.

### 2.4 Statistical Analysis

Sample size was estimated for the primary agreement analyses of the registered parent study, including lateral and non‐lateral body positions. The calculation was based on expected hydrostatic pressure differences derived from CT‐based geometric estimates of right atrial displacement relative to external reference points. Vertical distance differences were converted to pressure differences using standard hydrostatic conversion principles. Assuming a clinically relevant difference of 2 mmHg, a standard deviation of 2 mmHg, and 90% power, at least 13 patients were required. Continuous data are presented as mean ± standard deviation (SD) or median (range), and categorical variables as counts and percentages.

Agreement between RAP and CVP was assessed with Bland–Altman analysis (bias ±1.96 SD) [13], including 95% confidence intervals (CI) for bias and limits of agreement (LoA). Systematic differences were tested with the Wilcoxon signed‐rank test for paired data.

For clinical interpretation, agreement was additionally expressed as the proportion of paired RAP–CVP values within ±2 and ±2.5 mmHg, thresholds considered clinically relevant for invasive haemodynamic agreement analyses in prior critical care literature [14, 15]. Mean absolute error (MAE) and median absolute error (MedAE) were also calculated. Reliability of RAP–CVP agreement was assessed using the intraclass correlation coefficient (ICC, Shrout–Fleiss two‐way random model) [16].

To identify the optimal external reference point in lateral positions, mean RAP–CVP differences were recalculated post hoc after applying fixed hydrostatic offsets (0–5 cm equivalents) to the recorded CVP values.

Changes in RAP, MAP, and pulse pressure between body positions were analysed using the Wilcoxon signed‐rank test for paired data. For these analyses, results are presented as mean differences with 95% confidence intervals (CI) and corresponding *p*‐values. Pulse pressure analyses excluded the patient with atrial fibrillation.

## 3 Results

### 3.1 Patient Characteristics

A total of 27 patients were consecutively screened and 16 were finally included in the analysis. Out of 27 patients, 11 were excluded: Two patients because their relatives did not give consent, and nine patients due to measurement‐related issues. Seven were excluded because the predefined catheter offset stability criterion (> 0.5 mmHg) was exceeded, and two because of failed insertion or suboptimal positioning of the solid‐state catheter (Figure S4). Mean (range) age was 67.9 (41–83) years and mean (SD) BMI was 24.0 (6.1) kg/m^2^ (Table 1). No complications related to catheterisation or repositioning occurred.

**TABLE 1:** Patient characteristics.

Characteristics	Total (*n* = 16)
Age (year)	67.9 (11.5)
Female sex	4 (25.0)
Height (cm)	172.1 (8.7)
Weight (kg)	84.3 (25.1)
Body mass index (kg/m^2^)	24 (6.1)
Antero‐posterior diameter (cm)	22.3 (3.6)
SOFA score	11.4 (2.9)
Diagnosis	
Cardiac arrest	5 (31.2)
Aortic surgery (BEVAR/TEVAR)	4 (25.0)
Liver transplantation	2 (12.5)
Septic shock	3 (18.8)
Other	2 (12.5)
Left ventricular ejection fraction (%)	45.3 (13.2)
Baseline ventilator settings	
PEEP	10 (2.2)
Pmean/peak pressure	13.8 (2.8)/20.8 (2.7)
Respiratory rate	17.2 (2.3)
FiO_2_ (%)	39.7 (11.8)
Baseline haemodynamic data	
Mean arterial pressure (mm Hg)	83.6 (13.6)
Right atrial pressure (mm Hg)	8.5 (1.5)
Norepinephrine dose (μg/kg/min)	0.25 (0.21)
Atrial fibrillation	1 (6.3)

*Note:* Binary characteristics are reported as *n* (%) and continuous characteristics as mean (SD).

### 3.2 Right Lateral Position

**FIGURE 1:**
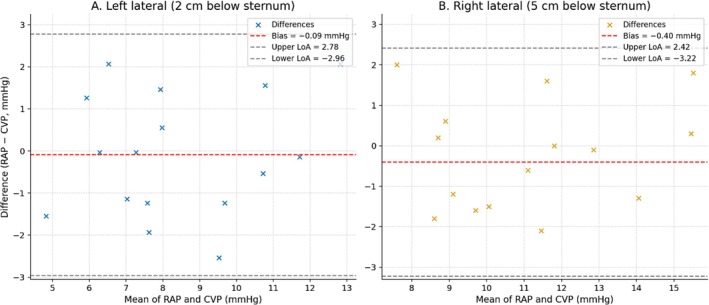
Bland–Altman plot showing RAP−CVP agreement in the left lateral (45°) position (A) with the transducer 2 cm below the mid‐sternum and the right lateral (45°) position (B) with the transducer 5 cm below the mid‐sternum. Mean RAP–CVP difference is shown as the dashed line; dotted lines indicate 95% limits of agreement.

With the CVP transducer levelled 5 cm below the mid‐sternum, CVP slightly overestimated RAP. The mean bias (RAP–CVP) was −0.4 mmHg (95% CI −1.3 to 0.6), with limits of agreement from −3.2 to +2.4 mmHg (Figure 1). Reliability was good (ICC = 0.85; Table 2). Coverage analysis showed that 87.5% of paired measurements were within ±2 mmHg and 93.8% within ±2.5 mmHg of RAP, with a mean absolute error of 1.2 mmHg and median absolute error of 1.4 mmHg (Table S1). Stepwise adjustment showed no meaningful improvement, as the offset was negligible, corresponding to approximately 0.5 cmH_2_O. Thus, the CT‐derived 5‐cm reference point was optimal in this position (Figure S5).

**TABLE 2:** Agreement between directly measured right atrial pressure (RAP) and central venous pressure (CVP) in 45° lateral positions.

Position	Zero level	Bias	LoA	ICC
Right lateral	5	−0.4	−3.2 to +2.4	0.85
Right lateral	3	+1.9	−0.9 to +4.7	0.69
Right lateral	0	+4.1	+1.3 to +6.9	0.39
Left lateral	5	−2.2	−5.0 to +0.7	0.58
Left lateral	3	−0.7	−3.5 to +2.2	0.79
Left lateral	2	−0.1	−3.0 to +2.8	0.82
Left lateral	0	+1.6	−1.3 to +4.4	0.60

*Note:* Bias is expressed as RAP–CVP; negative values indicate CVP overestimation of RAP. In the right lateral position, additional intermediate levels between 3 and 5 cm below the mid‐sternum were not evaluated because the 5‐cm reference level already showed smaller bias than the 3‐cm level. In the left lateral position, an additional 2‐cm level was evaluated to identify the reference level with the smallest bias.

Abbreviations: ICC, intraclass correlation coefficient (two‐way random effects, absolute agreement); LoA, limits of agreement (bias ±1.96 SD).

### 3.3 Left Lateral Position

When levelled 5 cm below the mid‐sternum, CVP systematically overestimated RAP. The mean bias (RAP–CVP) was −2.2 mmHg (95% CI −3.3 to −1.1), with limits of agreement from −5.0 to +0.7 mmHg (Figure 1). Reliability was moderate (ICC = 0.58; Table 2). Only 50% of paired measurements were within ±2 mmHg and 69% within ±2.5 mmHg of RAP, with a mean absolute error of 2.2 mmHg and median absolute error of 2.1 mmHg (Table S1). Mathematical adjustment of the transducer height indicated that raising the reference point by 3 cm reduced bias to approximately −0.1 mmHg and narrowed limits of agreement to −3.0 to +2.8 mmHg, with improved coverage. Thus, approximately 2 cm below the mid‐sternum was the optimal reference point in this dataset (Figure S5).

### 3.4 Correlation Analyses

Exploratory Spearman analyses showed that in the right lateral position, both AP chest diameter (*ρ* = −0.61, *p* = 0.012) and BMI (*ρ* = −0.52, *p* = 0.040) demonstrated significant negative correlations with RAP–CVP differences, indicating that larger body habitus was associated with more negative RAP–CVP differences and greater CVP overestimation of RAP (Table S2; Figures S6 and S7). LVEF showed no significant association (*ρ* = −0.34, *p* = 0.201). In the left lateral position, no significant associations were observed between RAP–CVP differences and BMI, AP diameter, or LVEF (all *p* > 0.20; Table S2).

### 3.5 Changes in RAP Between Positions

**FIGURE 2:**
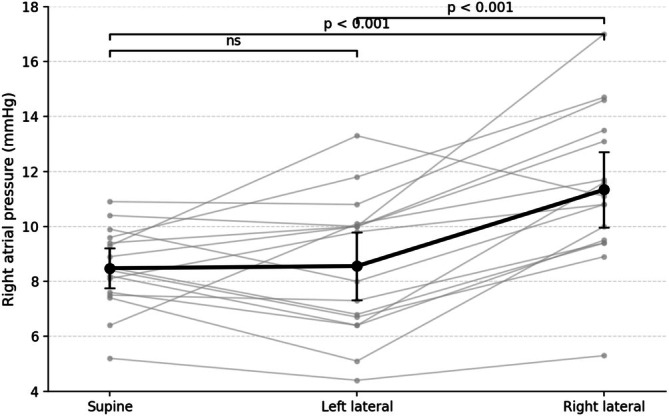
Right atrial pressure (RAP) across body positions. Thin grey lines represent individual patients; black markers and error bars indicate mean and 95% confidence intervals. RAP increased in the right lateral position compared with both supine and left lateral positions, whereas no difference was observed between supine and left lateral positions.

RAP in the right lateral position (11.3 ± 2.8 mmHg) was significantly higher than in supine (Δ +2.9 mmHg, *p* < 0.001) and left lateral (Δ +2.8 mmHg, *p* < 0.001) positions (Figure 2). There was no difference in RAP between supine (8.5 ± 1.5 mmHg) and left lateral positions (8.6 ± 2.5 mmHg; *p* = 0.78).

### 3.6 Exploratory Haemodynamic Changes

Pulse pressure was lower in the right lateral position compared with both supine and left lateral positions (*n* = 15; one patient excluded due to atrial fibrillation‐related beat‐to‐beat variability; Wilcoxon *p* = 0.048 and *p* = 0.022, respectively). Mean arterial pressure showed a non‐significant trend toward lower values in the right lateral position compared with the left lateral position (*n* = 16; Wilcoxon *p* = 0.13; Table 3).

**TABLE 3:** Haemodynamic parameters by body position.

Parameter	Supine (*n* = 16)	Left lateral 45° (*n* = 16)	Right lateral 45° (*n* = 16*)	*p* ^a^
RAP (mmHg)	8.5 ± 1.5	8.6 ± 2.5	11.3 ± 2.8	< 0.001^b^
MAP (mmHg)	87.7 ± 14.4	89.3 ± 15.8	86.6 ± 14.0	0.13^c^
Pulse pressure (mmHg)	78.7 ± 17.4	80.7 ± 16.0	74.1 ± 17.8	0.022^d^

*Note:* Data are mean ± SD.

* Pulse pressure was available in 15 patients (one excluded due to atrial fibrillation‐related beat‐to‐beat variability).

^a^ Wilcoxon signed‐rank test for paired comparisons.

^b^ Right lateral versus supine.

^c^ Right lateral versus left lateral.

^d^ Right lateral versus left lateral (*n* = 15).

## 4 Discussion

This study is the first to compare RAP, measured with a solid‐state catheter in the mid‐right atrium, with CVP referenced to external landmarks in the lateral position in critically ill mechanically ventilated patients. We found that using the CT‐derived reference point 5 cm below the mid‐sternum may systematically overestimate RAP and lead to misinterpretation of preload or venous congestion in the left lateral position. Adjusting the reference level to approximately 2 cm below the sternum markedly improved agreement with directly measured RAP across body positions.

RAP was higher in the right lateral than in supine or left lateral positions, consistent with prior experimental data [17]. This likely reflects combined geometric and cardiopulmonary effects. In the right lateral position, the right atrium is displaced deeper relative to the sternum [18], increasing hydrostatic pressure relative to a fixed external reference. Mechanical ventilation may further accentuate pleural pressure differences between dependent and non‐dependent hemithoraces [19, 20], increasing external pressure on the right heart. According to Guyton physiology, elevation of RAP in the dependent hemithorax would reduce the venous return gradient unless compensated by increased mean systemic filling pressure [1]. Together, these findings suggest that lateral positioning may influence right heart loading conditions through physiological mechanisms beyond hydrostatic measurement effects alone.

Measurements were obtained 3 min after each repositioning. This interval likely allowed partial haemodynamic compensation, including baroreflex‐mediated increases in venous tone and stressed volume, which may elevate mean systemic filling pressure [1, 21]. Such compensatory mechanisms may partially preserve mean arterial pressure despite a reduction in venous return. Accordingly, the modest change in MAP observed in the right lateral position may underestimate the immediate haemodynamic impact of lateral tilt, whereas the persistent increase in RAP and reduction in pulse pressure suggest ongoing alterations in right heart loading conditions.

Our findings may help explain discrepancies in a previous study by Bein et al., who reported unchanged CVP between supine and lateral positions despite changes in cardiac index [22]. Transducer levelling at the tracheal carina may introduce hydrostatic bias in lateral positions because the right atrium shifts relative to fixed external landmarks [23]. Consistent with prior imaging and experimental studies [17, 18, 23, 24], our findings suggest that lateral tilt alters both atrial geometry and intracardiac pressures, whereas head‐of‐bed elevation has less effect on atrial position relative to the sternum [25]. Sedation and controlled mechanical ventilation may also have reduced variability by minimising intrathoracic pressure fluctuations [26, 27, 28].

Since critically ill patients frequently remain in lateral positions for prolonged periods during pressure injury prevention, respiratory management, and routine nursing care, these findings may have implications for interpretation of venous congestion and right‐sided filling pressures in haemodynamically vulnerable patients, particularly those with impaired right ventricular function or vasopressor dependence.

Exploratory analyses suggested that higher BMI and AP chest diameter were associated with RAP–CVP differences in the right lateral position, although no such associations were observed in the left lateral position. This finding raises the possibility that larger body habitus amplifies hydrostatic discrepancies when the right atrium is displaced deeper into the thorax. No correlation was observed with LVEF, supporting the hypothesis that positional effects arise primarily from geometric and pleural mechanics rather than intrinsic cardiac function. These observations remain hypothesis‐generating given the limited sample size.

Although this study was not designed to assess cardiac output, the observed position‐dependent changes in RAP raise important physiological questions. Future studies should evaluate whether true increases in RAP during lateral positioning—measured with accurate levelling—translate into measurable changes in venous return and cardiac output in critically ill patients. Such studies would help clarify whether previously reported position‐related changes in cardiac output were masked or distorted by systematic levelling errors, and whether lateral positioning can be used as a deliberate haemodynamic intervention rather than merely a nursing manoeuvre.

### 4.1 Strengths and Limitations

Key strengths include conduct in a real‐world ICU cohort with diverse anthropometrics and cardiac characteristics, a prospective positioning protocol, and high‐fidelity RAP measurement with a solid‐state catheter verified by waveform and echocardiography. This approach minimised systematic measurement error and enhances the clinical applicability of our findings.

Although the study was adequately powered for the primary endpoint, the limited sample size restricted subgroup analyses and may limit generalisability. The fixed sequence of positioning may have introduced carry‐over effects or haemodynamic drift over time. Although measurements were obtained after a stabilisation period in each position, randomisation of positioning order could have strengthened the study design. Catheter position was confirmed by waveform analysis and selective echocardiography, making major misclassification unlikely, although ultrasound was not feasible in every position. The protocol examined only a 45° lateral tilt with the head‐of‐bed 0°. Hydrostatic relationships may differ at other tilt angles or with head elevation, and mechanical ventilation parameters (e.g., higher PEEP) could further influence RAP. Finally, the cohort partially overlaps with concurrent analyses from the same registered protocol investigating non‐lateral body positions, although the present analysis was predefined to specifically evaluate lateral positioning.

## 5 Conclusion

In mechanically ventilated ICU patients, both right atrial pressure and the accuracy of central venous pressure measurement are influenced by body position. Optimal external reference levels differed between lateral positions, with approximately 5 cm below the mid‐sternum providing the closest agreement with directly measured right atrial pressure in the right lateral position, and approximately 2 cm below the mid‐sternum minimising systematic bias in the left lateral position. In addition, right atrial pressure increased in the right lateral position, suggesting that lateral positioning may influence cardiopulmonary physiology beyond hydrostatic measurement effects alone.

## Author Contributions

C.S. contributed to study design, data collection, data analysis and interpretation, and manuscript drafting. L.J., C.J.S., P.P., S.O., and J.O. contributed to study design, data analysis and interpretation, and manuscript drafting. A.I., J.H., and N.V. contributed to study design, data collection, and manuscript revision. All authors read and approved the final manuscript.

## Funding

This work was supported by grants from the Swedish state under the agreement between the Swedish government and the county councils, the ALF‐agreement (ALFGGBG‐70150), The Local Research and Development Council Gothenburg and Södra Bohuslän (VGFOUGSB‐982802), and Sahlgrenska University Hospital Foundations (SU‐984490).

## Consent

The authors have nothing to report.

## Conflicts of Interest

The authors declare no conflicts of interest.

## Supporting information


**Figure S1:** Red pressure curve ‐RAP, blue pressure curve—CVP, green pressure curve—arterial pressure. Initial right ventricular pressure curve and after slight withdrawal of the solid‐state catheter, RAP curve indicating correct positioning of the catheter.
**Figure S2:** Cardiac ultrasound during insertion of the solid‐state catheter. The solid‐state catheter is visible in the right atrium.
**Figure S3:** After NaCl flush, the catheter shows acceptable offset (−0.29 mmHg).
**Figure S4:** Study screening and inclusion flowchart. A total of 27 patients were consecutively assessed for eligibility. Eleven patients were excluded: two due to lack of next‐of‐kin consent and nine due to measurement‐related issues (seven with solid‐state catheter offset > 0.5 mmHg and two with failed insertion or improper catheter positioning). Sixteen patients were included in the final analysis.
**Table S1:** Agreement and reliability metrics for RAP versus CVP in the lateral positions.
**Table S2:** Spearman rank correlations between anthropometric/cardiac variables and RAP–CVP differences.
**Figure S5:** Optimal external reference levels for CVP measurement in left and right lateral positions. This figure is intended as an illustrative schematic to highlight study findings and does not represent an exact anatomical reconstruction. Parts of figure created with the assistance of AI‐based image generation (ChatGPT, OpenAI) and edited using Microsoft Designer.
**Figure S6:** Scatterplot illustrating the relationship between body mass index (BMI) and the difference between right atrial pressure (RAP) and central venous pressure (CVP) in the 45° right lateral position. Each point represents an individual patient. A positive RAP–CVP difference indicates underestimation of RAP by CVP, whereas a negative difference indicates overestimation of RAP by CVP. Spearman's rank correlation demonstrated a significant negative association (*ρ* = −0.52, *p* = 0.040), indicating that higher BMI was associated with more negative RAP–CVP differences and greater CVP overestimation of RAP. The solid red line represents the fitted linear regression trend.
**Figure S7:** Scatterplot showing the relationship between anteroposterior (AP) chest diameter and the difference between right atrial pressure (RAP) and central venous pressure (CVP) in the right lateral position. Each point represents an individual patient. A positive RAP–CVP difference indicates underestimation of RAP by CVP, whereas a negative difference indicates overestimation of RAP by CVP. Spearman's rank correlation demonstrated a significant negative association (*ρ* = −0.61, *p* = 0.012), indicating that larger AP diameter was associated with more negative RAP–CVP differences and greater CVP overestimation of RAP. The red line represents the fitted linear trend.

## Data Availability

The data that support the findings of this study are available on request from the corresponding author. The data are not publicly available due to privacy or ethical restrictions.
