# Supplementary material for: Central Venous Pressure Referencing in the Lateral Position: Comparison With Direct Right Atrial Pressure in Mechanically Ventilated ICU Patients
Source: Acta Anaesthesiol Scand. 2026 Jun 12;70(6):e70280. doi: 10.1111/aas.70280 (PMC13263127; doi:10.1111/aas.70280)
Supplement: Supplementary file 1 — Figure S1: Red pressure curve ‐RAP, blue pressure curve—CVP, green pressure curve—arterial pressure. Initial right ventricular pressure curve and after slight withdrawal of the solid‐state catheter, RAP curve indicating correct positioning of the catheter. Figure S2: Cardiac ultrasound during insertion of the solid‐state catheter. The solid‐state catheter is visible in the right atrium. Figure S3: After NaCl flush, the catheter shows acceptable offset (−0.29 mmHg). Figure S4: Study screening and inclusion flowchart. A total of 27 patients were consecutively assessed for eligibility. Eleven patients were excluded: two due to lack of next‐of‐kin consent and nine due to measurement‐related issues (seven with solid‐state catheter offset > 0.5 mmHg and two with failed insertion or improper catheter positioning). Sixteen patients were included in the final analysis. Table S1: Agreement and reliability metrics for RAP versus CVP in the lateral positions. Table S2: Spearman rank correlations between anthropometric/cardiac variables and RAP–CVP differences. Figure S5: Optimal external reference levels for CVP measurement in left and right lateral positions. This figure is intended as an illustrative schematic to highlight study findings and does not represent an exact anatomical reconstruction. Parts of figure created with the assistance of AI‐based image generation (ChatGPT, OpenAI) and edited using Microsoft Designer. Figure S6: Scatterplot illustrating the relationship between body mass index (BMI) and the difference between right atrial pressure (RAP) and central venous pressure (CVP) in the 45° right lateral position. Each point represents an individual patient. A positive RAP–CVP difference indicates underestimation of RAP by CVP, whereas a negative difference indicates overestimation of RAP by CVP. Spearman's rank correlation demonstrated a significant negative association (ρ = −0.52, p = 0.040), indicating that higher BMI was associated with more negative RAP–CVP dif [file AAS-70-0-s001.docx]

**Supplementary file.**

**Central venous pressure referencing in the lateral position: comparison with direct right atrial pressure in mechanically ventilated ICU patients**

**Catheter Positioning and Stability Verification**To ensure accurate and reproducible RAP measurements, the solid-state catheter was securely fixed within the lumen of the central venous catheter (CVC) to minimise displacement. The head, neck, and arms were maintained in a neutral position during all measurements to reduce the risk of catheter migration with positional changes.

Catheter tip location was verified using transthoracic echocardiography whenever feasible. When anatomical variation or suboptimal acoustic windows prevented adequate visualisation, alternative echocardiographic views were employed. In each case, the image providing the clearest visualisation of the catheter tip was selected, though only the most illustrative image was retained for documentation. Tip position was confirmed after positional changes in the first five patients and, in the remaining participants, repeat ultrasound was performed only if changes in RAP waveform morphology suggested possible displacement.

In addition to ultrasound, pressure waveform analysis was used to confirm intravascular position and proximity to the tricuspid valve. The catheter was advanced until a right ventricular pressure waveform appeared, then slowly withdrawn until a right atrial pressure waveform was obtained, indicating entry into the right atrium. The tip was then retracted an additional 1 cm to position it near the mid-right atrium.

**Procedure**

Solid-state catheter insertion and verification were performed by experienced anaesthesiologists trained in invasive haemodynamic monitoring and cardiac ultrasound, supported by a biomedical engineer and intensive care nurses.

Measurements were obtained sequentially. All patients were first placed in the 45° left lateral orientation using a positioning pillow. After completion of recordings in this position, they were consistently repositioned to the 45° right lateral orientation. A standardised rest period of three minutes was observed after each repositioning before data collection to allow for haemodynamic stabilisation. This sequence was applied uniformly to all participants to ensure methodological consistency.

**Insertion, zeroing and measurements**

1. The Millar Micro-Cath was zeroed against atmospheric pressure.
2. The catheter was pre-soaked in a 37°C NaCl solution.
3. After the catheter was removed from the NaCl solution and exposed to air, it was re-zeroed if there is any drift from zero.
4. The catheter was inserted via the medial lumen of the central venous catheter (CVC); when right ventricular pressure was recorded, the catheter was withdrawn until RAP pressure curve appears (Fig. S1). The catheter was then retracted an additional 1 cm to position the tip near the mid-right atrium.


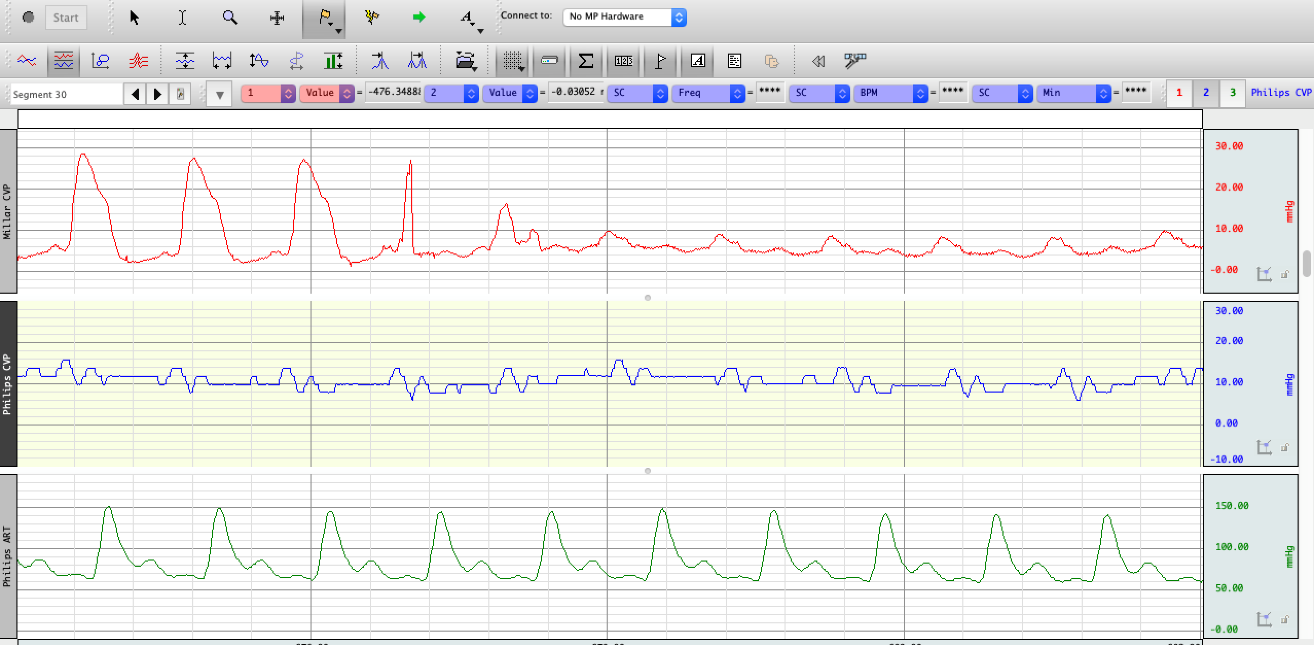


**Fig. S1.** Red pressure curve -RAP, blue pressure curve – CVP, green pressure curve – arterial pressure. Initial right ventricular pressure curve and after slight withdrawal of the solid-state catheter, RAP curve indicating correct positioning of the catheter.

1. The catheter tip position was verified using cardiac ultrasound (Fig. S2).


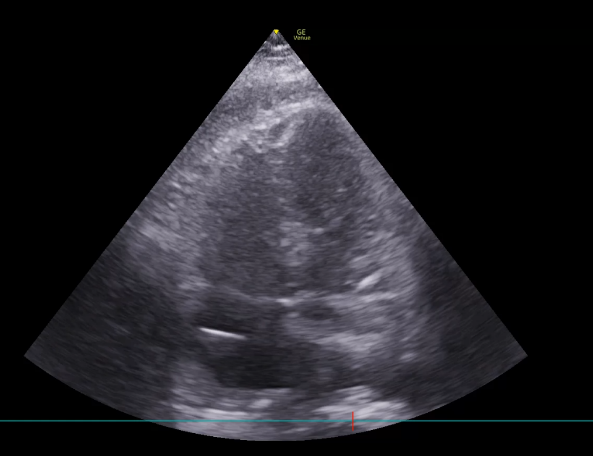


**Fig. S2** Cardiac ultrasound during insertion of the solid-state catheter. The solid-state catheter is visible in the right atrium.

1. CVP was measured through the distal lumen of the CVC. The CVP and arterial pressure sensors were zeroed against atmospheric pressure.
2. In both lateral positions, the pressure transducer was aligned with 5 cm below the mid-sternum in the fourth intercostal space. Levelling was made with a modified water levelling device.
3. The mean CVP and RAP values for one cardiac cycle were recorded simultaneously during the end-expiratory phase, 3 minutes after each position change.
4. The Micro-Cath was removed from the CVC and flushed with a 20 cc NaCl syringe.
5. The Micro-Cath offset is recorded (Fig. S3).


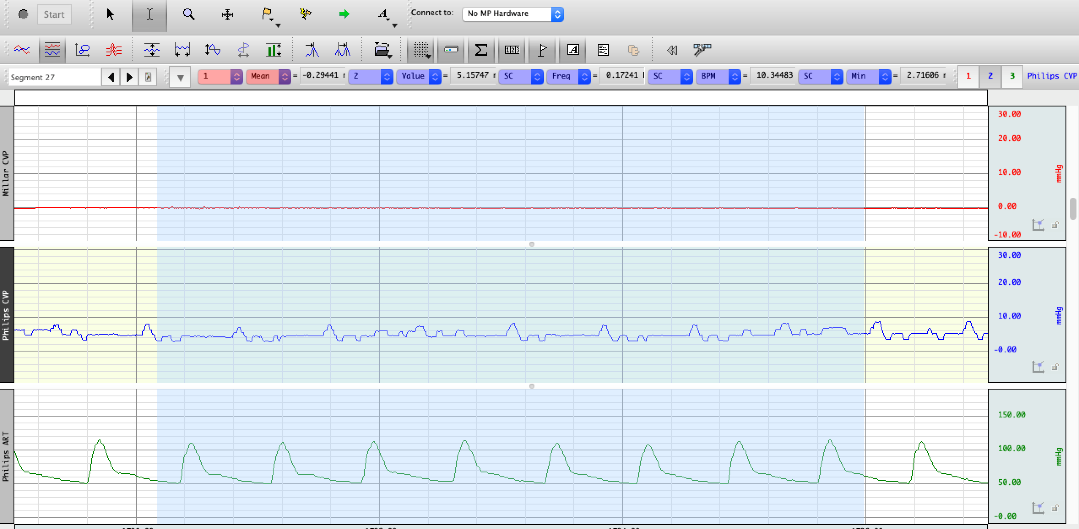


**Fig. S3** After NaCl flush, the catheter shows acceptable offset (–0.29 mmHg)


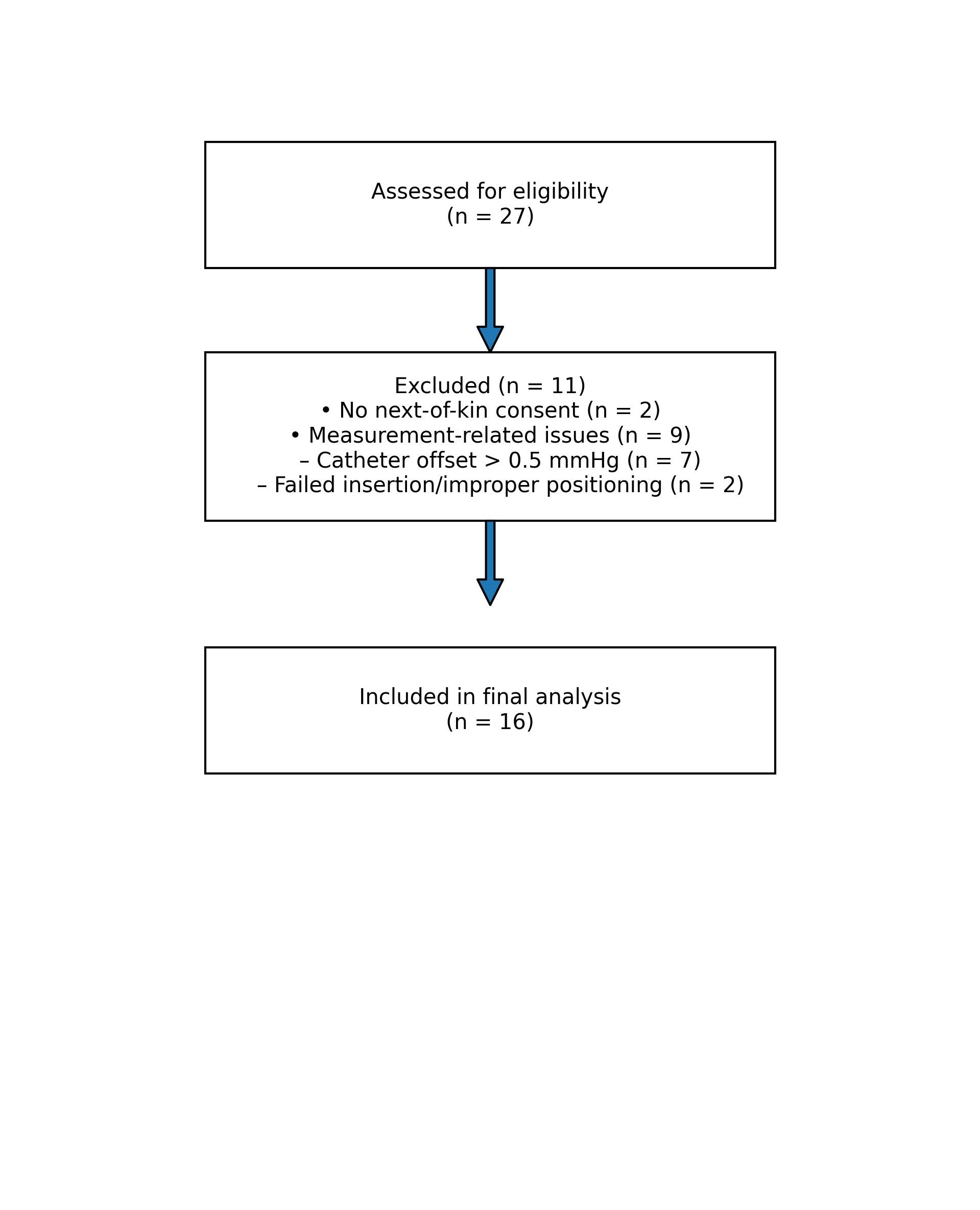


**Fig. S4.** Study screening and inclusion flowchart. A total of 27 patients were consecutively assessed for eligibility. Eleven patients were excluded: two due to lack of next-of-kin consent and nine due to measurement-related issues (seven with solid-state catheter offset > 0.5 mmHg and two with failed insertion or improper catheter positioning). Sixteen patients were included in the final analysis.

**Table S1.** Agreement and reliability metrics for RAP versus CVP in the lateral positions

| **Position** | **Zero level (cm below sternum)** | **Bias (mmHg)** | **95% CI of bias (mmHg)** | **SD (mmHg)** | **LoA (mmHg)** | **MAE (mmHg)** | **MedAE (mmHg)** | **ICC(2,1) [95% CI]** |
| --- | --- | --- | --- | --- | --- | --- | --- | --- |
| Right lateral 45° | 5 | −0.40 | −−1.17 to +0.37 | 1.44 | −3.22 to +2.42 | 1.21 | 1.40 | 0.85 [0.72–0.91] |
| Right lateral 45° | 3 | +1.88 | +1.11 to +2.65 | 1.44 | −0.94 to +4.70 | 1.99 | 1.82 | 0.69 [0.47–0.82] |
| Right lateral 45° | 0 | +4.10 | +3.33 to +4.87 | 1.44 | +1.28 to +6.92 | 4.08 | 4.03 | 0.39 [0.21–0.53] |
| Left lateral 45° | 5 | −2.15 | −2.93 to −1.37 | 1.46 | −5.02 to +0.72 | 2.19 | 2.15 | 0.58 [0.35–0.72] |
| Left lateral 45° | 3 | −0.67 | −1.45 to +0.11 | 1.46 | −3.54 to +2.20 | 1.70 | 1.53 | 0.79 [0.61–0.89] |
| Left lateral 45° | 2 | −0.05 | −0.83 to +0.73 | 1.46 | −2.92 to +2.82 | 1.20 | 1.25 | 0.82 [0.61–0.89] |
| Left lateral 45° | 0 | +1.55 | +0.77 to +2.33 | 1.46 | −1.32 to +4.42 | 1.70 | 1.53 | 0.60 [0.39–0.83] |

*Bias is defined as RAP − CVP. Negative values indicate overestimation of RAP by CVP, whereas positive values indicate underestimation of RAP by CVP. The 95% confidence intervals for bias were calculated directly. LoA, limits of agreement calculated as bias ± 1.96 × SD; MAE, mean absolute error; MedAE, median absolute error; ICC(2,1), two-way random-effects, single-measure intraclass correlation coefficient for absolute agreement.* *In the right lateral position, additional intermediate levels between 3 and 5 cm below the mid-sternum were not evaluated because the 5-cm reference level already showed smaller bias than the 3-cm level. In the left lateral position, an additional 2-cm level was evaluated to identify the reference level with the smallest bias.*

# Table S2. Spearman rank correlations between anthropometric/cardiac variables and RAP–CVP differences

| **Variable** | **Right lateral (ρ, p)** | **Left lateral (ρ, p)** |
| --- | --- | --- |
| AP diameter | ρ = −0.61, p = 0.012 | ρ = −0.07, p = 0.79 |
| BMI | ρ = −0.52, p = 0.040 | ρ = −0.11, p = 0.70 |
| LVEF (%) | ρ = −0.34, p = 0.20 | ρ = +0.00, p = 0.99 |

*Spearman rank correlation coefficients (ρ) with corresponding p-values between anthropometric/cardiac variables and the difference between right atrial pressure (RAP) and central venous pressure (CVP) in right and left lateral positions.*

**
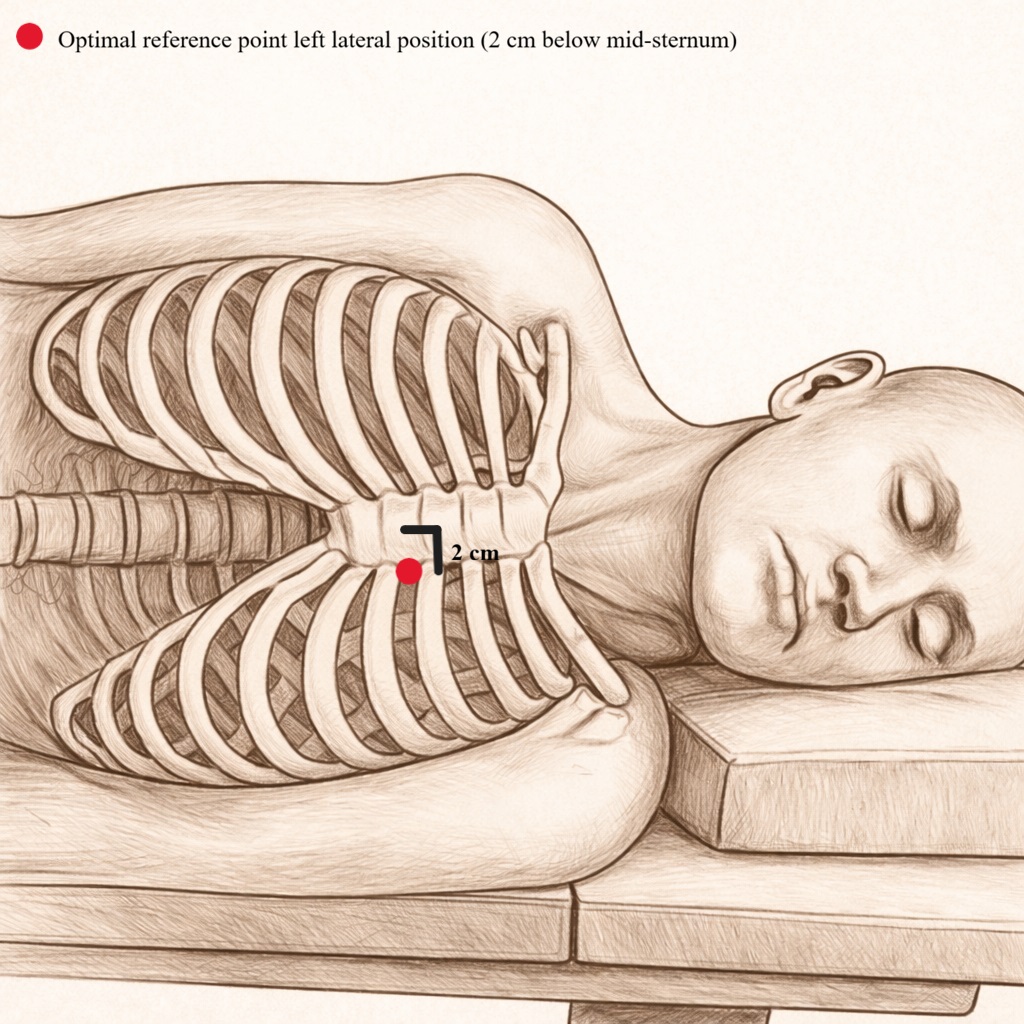

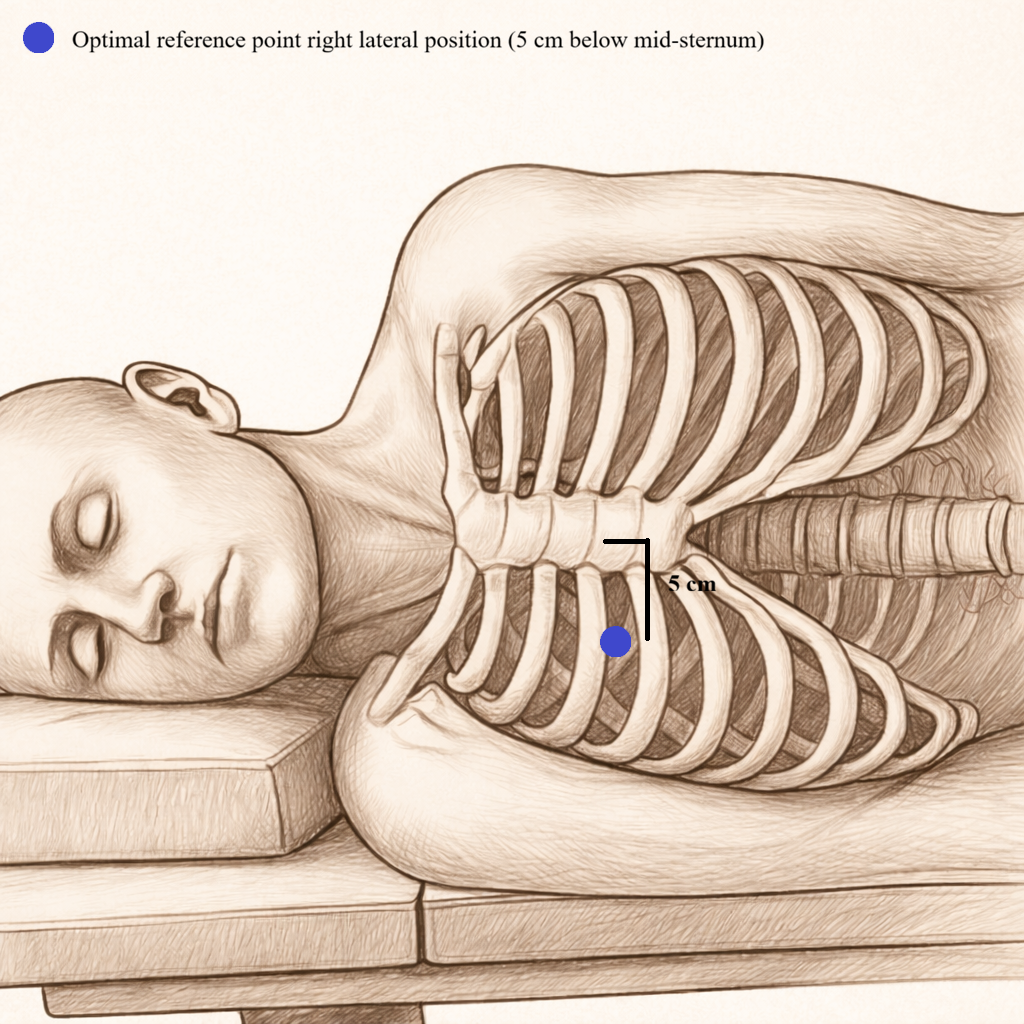
**

**Fig. S5.** Optimal external reference levels for CVP measurement in left and right lateral positions. This figure is intended as an illustrative schematic to highlight study findings and does not represent an exact anatomical reconstruction. Parts of figure created with the assistance of AI-based image generation (ChatGPT, OpenAI) and edited using Microsoft Designer.


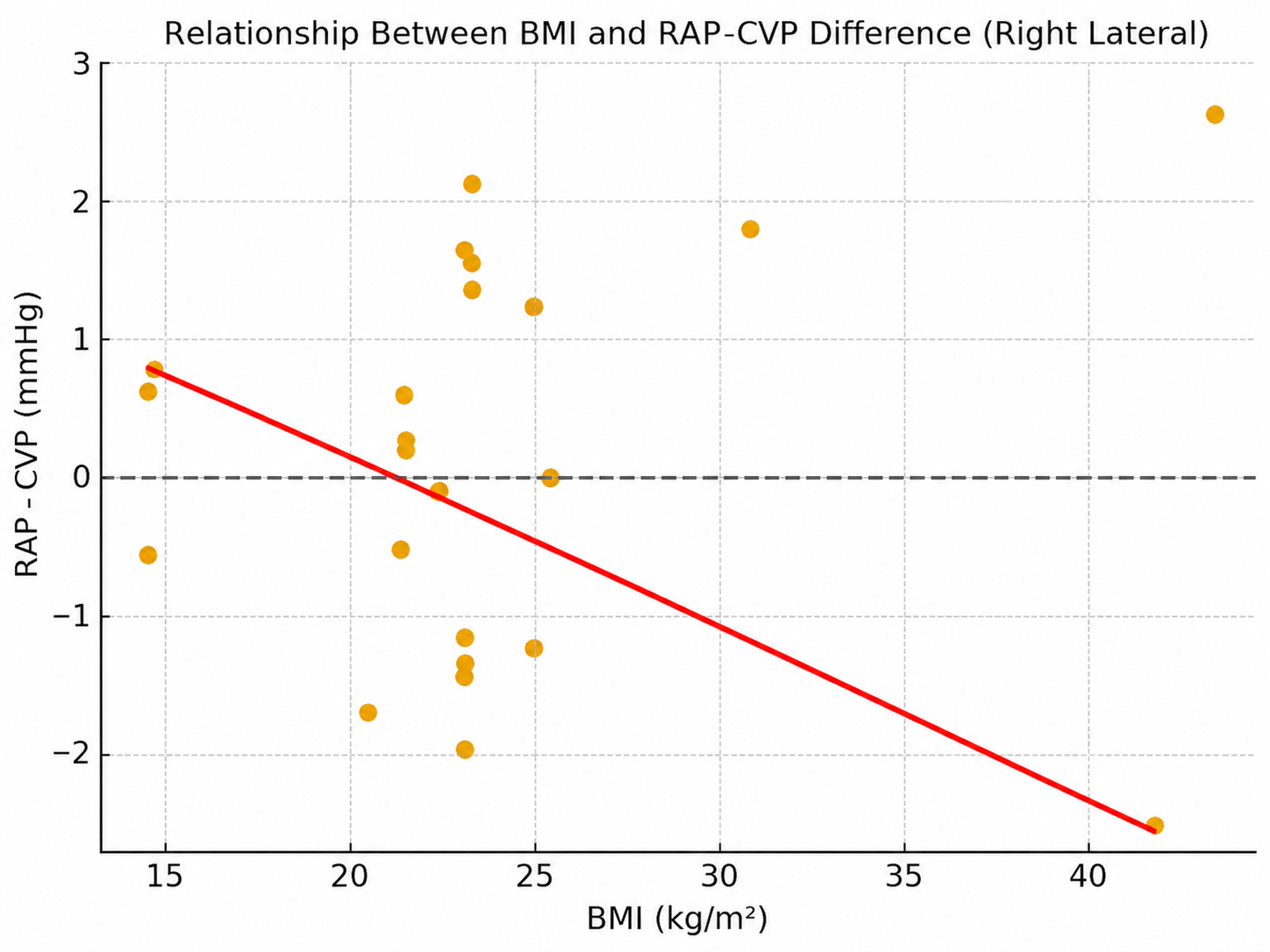


**Fig. S6.** Scatterplot illustrating the relationship between body mass index (BMI) and the difference between right atrial pressure (RAP) and central venous pressure (CVP) in the 45° right lateral position. Each point represents an individual patient. A positive RAP–CVP difference indicates underestimation of RAP by CVP, whereas a negative difference indicates overestimation of RAP by CVP. Spearman’s rank correlation demonstrated a significant negative association (ρ = −0.52, p = 0.040), indicating that higher BMI was associated with more negative RAP–CVP differences and greater CVP overestimation of RAP. The solid red line represents the fitted linear regression trend.
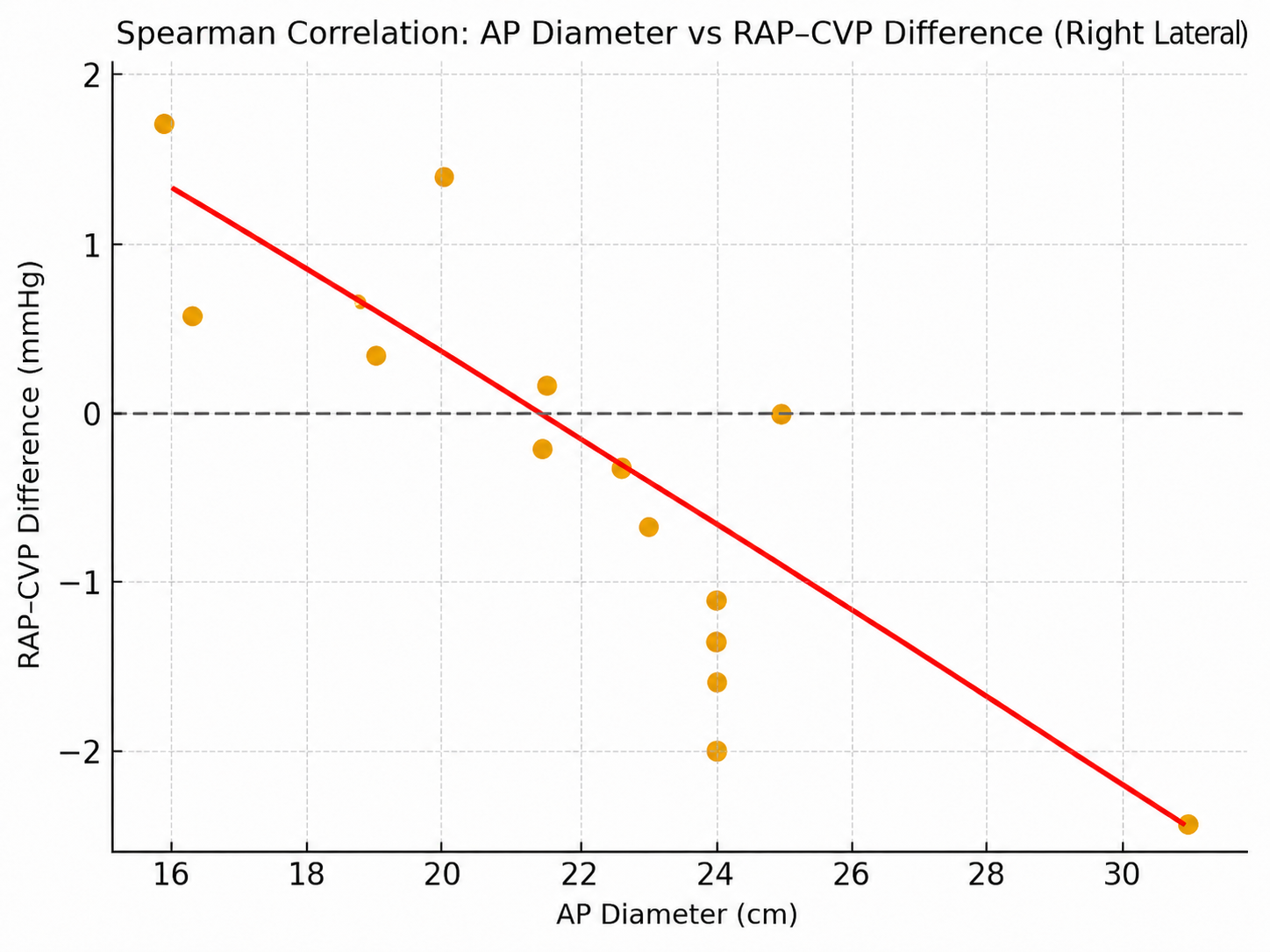


**Fig. S7.** Scatterplot showing the relationship between anteroposterior (AP) chest diameter and the difference between right atrial pressure (RAP) and central venous pressure (CVP) in the right lateral position. Each point represents an individual patient. A positive RAP–CVP difference indicates underestimation of RAP by CVP, whereas a negative difference indicates overestimation of RAP by CVP. Spearman’s rank correlation demonstrated a significant negative association (ρ = −0.61, p = 0.012), indicating that larger AP diameter was associated with more negative RAP–CVP differences and greater CVP overestimation of RAP. The red line represents the fitted linear trend.
